# Genome factor and gene pleiotropy hypotheses in protein evolution

**DOI:** 10.1186/1745-6150-5-37

**Published:** 2010-05-24

**Authors:** Yanwu Zeng, Xun Gu

**Affiliations:** 1 School of Life Sciences, Fudan University, Shanghai 200433, China; 2 Department of Genetics, Developmental and Cell Biology, Iowa State University, Ames, IA 50011, USA

## Abstract

The debate of genomic correlations between sequence conservation, protein connectivity, gene essentiality and gene expression, has generated a number of new hypotheses that are challenging the classical framework of molecular evolution. For instance, the translational selection hypothesis claims that the determination of the rate of protein evolution is the protein stability to avoid the misfolding toxicity. In this short article, we propose that gene pleiotropy, the capacity for affecting multiple phenotypes, may play a vital role in molecular evolution. We discuss several approaches to testing this hypothesis.

**Reviewers:**

This article was reviewed by Dr Eugene Koonin, Dr Arcady Mushegian and Dr Claus Wilke.

## Background

A high controversy in evolutionary genomics is around the pervasive yet weak genomic correlations with the evolutionary rate of protein sequence [1,2]. Initially, the debate was about whether these weak correlations are statistically meaningful or are caused by the confound effects of gene expression [3-7]. Recently, the research focus is shifting to having a better understanding of the underlying mechanisms [8-17]. In spite of a number of controversial issues, the logic of this type of analysis remains roughly the same, that is, statistically establish the correlation between biologically factors and the evolutionary rate. These efforts have generated some interesting hypotheses, but it has been found difficult to be integrated to the theory of molecular evolution [17] based on several population genetics mechanisms and assumptions about the genotype-phenotype mapping. Hence, it is desirable further to explore how genome factors can affect the underlying mechanisms of molecular evolution.

The inverse relationship between functional importance and evolutionary rate, sometimes regarded as the first principle of molecular evolution, has played a fundamental role in the early development of the neutral theory [17]. High throughput functional genomics data have provided some opportunities to test this claim. For instance, over 30 years ago the relationship between gene dispensability and evolutionary rate was predicted [16]. While the genome-wide protein dispensability can be experimentally measured, people found there was indeed a significant yet weak correlation between protein dispensability and evolutionary rate in *S. cerevisiae *[3] and in bacteria species [18]. Here we use *the genome factor hypothesis *to represent all kinds of association studies between the rate of protein evolution and genome factors.

Another controversial example of the genome factor hypothesis is the yeast protein-protein interactions. Fraser et al. [4] demonstrated a negative correlation between the protein connectivity and the rate of protein sequence, that is, highly connected genes evolved slowly. However, re-analyses [7,18-22] suggested that the rate-interactivity correlation was rather weak, and the effect was largely caused by those network hubs (genes with high number of interactions) with low evolutionary rate. Besides, the rate-hub correlation has been heterogeneous among different datasets, probably sensitive to various technical treatments [6].

In contrast to above two examples, the evolutionary rate of protein sequence is strongly negatively correlated with the expression level, that is, highly expressed genes tend to evolve significantly slower than genes with low expression levels [19]. This has raised some hot debates whether the observed weak correlation between other genome factors (e.g., protein dispensability or hubs) and evolutionary rate could be confounded by the expression level [6]. Some of follow-up analyses have shown that these genomic correlations may remain statistically significant after controlling the expression effect at the phenomenological level, though understandably they became weaker [5]. Two studies [9,13] utilized the principal component analysis (PCA) to transform multiple correlated genomic variables into a smaller number of composite, independent canonical variables that account for a greater proportion of the variance of the evolutionary rate among genes. Drummond et al [9] concentrated on the substantial genomic data available in the yeast *S. cerevisiae*. Strikingly, they observed a single, dominant variable that determined the rate of protein evolution, which comprised variables related to expression level, codon adaptation index (CAI), and protein abundance. Their analyses suggested an intuitive-counter conclusion: Coding sequence evolution is primarily governed by the selection acting at the abundance level of gene expression and translation.

In short, the consensus conclusion is that, among currently available genome factors, expression level has the strongest contribution to the protein sequence evolution. In additional, a number of genome factors may have independent yet weak (some are very weak) contributions to the rate of protein evolution. However, we point out a potential risk of this consensus. Since a strong statistical association between two factors (expression level and rate of protein evolution) cannot infer the cause-effect relationship, we cannot empirically nor logically rule out the possibility that both are correlated with an unknown underlying mechanism, or, to another extreme, that coding sequence conservation determines the expression level. To address this issue, we have to explore the underlying mechanism.

## Presentation of the hypothesis

From the view of systems biology, the capacity of a gene to affect a number of phenotypic traits, known as gene pleiotropy, should play a major role in protein evolution [15,23-27]. As shown below, the problem is how this mechanism can be parameterized into the theory of molecular evolution, by the means of population genetics and genotype-phenotype mapping.

Gu [25,26] postulated that molecular evolution of a gene in a *K*-dimensional space of molecular phenotypes that represent the multi-functionality of the encoding protein. Under this framework, the functional dimensionality (*K*) can be viewed as a measure for gene pleiotropy. In particular, Gu [25] analyzed the pleiotropy model under the following assumptions: (*i*) *K *molecular phenotypes of the gene are under Gaussian-like stabilizing selection, indicating a single fitness optimum. Any deviation from the optimum is under the purifying selection. (*ii*) The fitness optimum of molecular phenotypes may shift randomly during the course of evolution, which may generate the process of *micro-adaptation*. And (*iii*) the distribution of mutational effects on molecular phenotypes follows a multivariate normal distribution. Under the theme of nearly-neutral evolution, *i.e.*, the stabilizing selection is strong and the micro-adaptation is weak, with close approximation the evolutionary rate can be written as follows(1)

where *v *is the mutation rate, *B*_0 _is the baseline selection intensity and *c *≈0.5772.

Therefore, the question for what is the main determinant of the rate of protein evolution may have different answers, depending on how to decipher the functional constraint imposed on the protein sequence. While the genome factor hypothesis is in attempt to establish the correlation between the biologically factor and the evolutionary rate, the gene pleiotropy hypothesis is to identify the degree of multi-functionality based on a statistical model of genotype-phenotype mapping. To be more specific, we have the followings:

*(i)	Gene pleiotropy (K) measures protein multi-functionality*: The parameter *K *relates to the functional importance of a gene in the up-level physiology and cellular networks [1,15,23,25,26]. Many genomic measures, such as protein-protein or protein-DNA interactivity, physiological processes involved, developmental stages, tissue expression broadness, etc., are biologically explanatory variables of the gene pleiotropy (*K*).

*(ii)	Baseline selection intensity (B*_0_) *involves many evolutionary mechanisms*: By contrast, the baseline selection intensity (*B*_0_) could be affected by various evolutionary forces. Symbolically, *B*_0 _can be written as *B*_0 _= -2*N*_*e*_(σ^2^_*m*_/σ^2^_*w*_)(1 - γ), where *N*_*e *_is the effective population size, σ^2^_*w *_is the strength of stabilizing selection on a single molecular phenotype (a smaller value means more stringent selection, and *vice versa*), σ^2^_*m *_is the mutational variance, and 0 < γ < 1 measures the effect of microadaptation. Hence, if a genome factor is correlated with *B*_0_, it could be the consequence of protein structure stability (a component of σ^2^_*w*_), mutability (σ^2^_*m*_) or micro-adaptation (γ). Apparently, such correlations are sensitive to the strength genetic drifts (*N*_*e*_) in different organisms (genomes).

## Testing the Hypothesis

In summary, evolutionary analyses from high throughput functional genomic datasets have formulated the genome factor hypothesis of molecular evolution. On the other hand, our studies [15,25,26] suggested that gene pleiotropy may be the main determinant for the evolutionary rate of protein sequence, called the gene pleiotropy hypothesis of molecular evolution. An immediate question is whether these two hypotheses are exclusive to each other. Actually, this is the most frequently asked question we have received since the publication of our work.

First, we emphasize that the genome factor hypothesis and the gene pleiotropy hypothesis represent two distinct approaches to deciphering the functional constraint of protein sequence evolution. In the case of genome factor hypothesis, the functional constraint is decomposed into a number of biological factors that can be experimentally measured at the genome level. The best scenario of this approach is to document in detail all biological factors that may influence the sequence conservation. Hence, the more we know about the genome complexity, the deeper we have the understanding of protein evolution. The difficulty of such data-driven approach is how to draw a clear evolutionary pattern, particularly when the biological factors are highly heterogeneous among genes and the genomic data are highly noisy. By contrast, the gene pleiotropy hypothesis of molecular evolution is mainly model-driven. That is, based on a statistical modeling of the genotype-phenotype association, the degree of gene pleiotropy, defined as the dimensionality (*K*) of genotype-phenotype space, can be integrated into the model of molecular evolution without requiring the biological detail.

**Figure 1:**
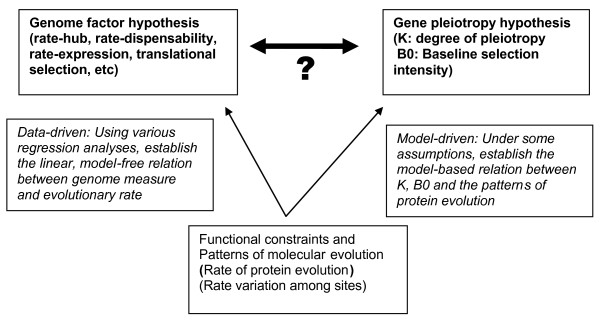
**A schematic illustration of the pleiotropy hypothesis and the genome factor hypothesis of protein evolution**.

In our view, the genome factor hypothesis and the gene pleiotropy hypothesis are complementary rather than exclusive, because two different interpretations of functional constraint may provide some new insights about how biological factors influence molecular evolution. Figure 1 illustrates how genomic factors are correlated with the gene pleiotropy (*K*) and/or the baseline selection intensity (*B*_0_). Suppose we have observed a statistically significant correlation between a particular genome factor and the rate of protein evolution. Under the theme of nearly-neutral evolution, one may further ask whether this genome factor is correlated with the gene pleiotropy (*K*) or the baseline selection intensity (*B*_0_). The biological significance is about the relative role between the functional importance in the biological system of a gene (gene pleiotropy) and the sequence-structure feature for the protein stability: Which one is the major determinant for the rate of protein evolution? Note that gene pleiotropy is a measure for the multiple roles of protein function in development and physiology. Meanwhile, the baseline selection intensity is the product of effective population size (*N*_*e*_) and other factors.

In particular, we discuss in some details about the translational selection hypothesis [12-14]. This theory demonstrates that one of major components in the functional constraint imposed in the protein sequence is the minimization of misfolding burden, or the sequence requirement to retain the capability to fold into the correct, functional protein structure. Broadly speaking, the effect of misfolding minimization is a combination of two factors: First, the proportion of (misfolded) proteins that need to be refolded or eliminated. Hence, the burden would be proportionally heavy for those highly-expressed genes. Consequently, genes coding for highly expressed proteins evolve slowly due to the stabilizing selection for the tolerance to translational errors that causes protein misfoldings. The second factor is the actual toxicity to the cell of some of the misfolded variants, which may be related to protein stability rather than protein abundance in the cell. As shown above, the baseline selection intensity (*B*_0_) combines some biochemical-biophysical properties of a gene (e.g., protein designability, stability and mutability) that may be attributed to the mutational variance σ^2^_*m*_, as well as the strength of stabilizing selection (σ^2^_*w*_). Together, the translational selection hypothesis predicts that under the translational selection hypothesis, the expression level should be correlated with the baseline selection intensity.

On the other hand, because optimal gene expression level is the basis for any protein to perform functions under a certain physiological condition, it is biologically reasonable to claims that a highly pleiotropic gene may have to maintain a relatively high level of gene expression. In this sense, we expected a positive correlation between gene pleiotropy (*K*) and the expression abundance. This argument reveals that the effect of gene expression on the rate of evolutionary rate, either abundance or broadness, may have the same underlying mechanism. According to Eq.(1), the rate-expression correlation could be the *B*_0_-mode (as predicted by the translational selection hypothesis), or the *K*-mode (basis for multi-functionality). The confound effect of expression on any *K*-related biological factors of protein functions is thus not too hard to understand.

Gu [25,26] developed a computational pipeline to estimate the effective gene pleiotropy (*K*_*e*_) from the phylogenetic analysis of protein sequences. Su et al [15] estimated the effective gene pleiotropy for 321 vertebrate genes, and found that a gene typically has *K*_*e *_= 6-7, as well as the baseline selection intensity *B*_0 _= 1-2. Moreover, we have recently analyzed several hundred genes from the yeast (five genomes) and *Drosophila *(12 genomes) and concluded that it seems to be a general pattern.

There are many biological factors that are related to the degree of gene pleiotropy such as number of enzymes substitutes, protein-protein or protein-DNA interactivity, physiological processes involved, different developmental stages, tissue expression broadness [1,2]. These genome factors may provide small, additive effects that together explain the extent of gene pleiotropy. We have two more comments. First, for a given gene, a limited number of biological factors can explain all nontrivial aspects of multi-functionality of the encoded protein. Second, contributions of these biological factors to the gene pleiotropy are highly heterogeneous among genes. Consequently, a specific type of high throughput technology such as protein-protein interactivity only measures the multi-functionality of some genes in the genome. Since many other genes may have trivial correlations with this genomic factor, one should expect there is only a weak genomic correlation *in prior*. Based on the limited dataset of vertebrate genes, Su et al. [15] observed the positive correlation of gene pleiotropy with the number of GO biological processes, as well as the expression broadness provides a biological basis for the sequence-based estimation of gene pleiotropy. To validate these observations, we recently have shown significant correlations between *K*_*e *_and protein-protein interactivity in the yeast, while there is no correlation between the baseline selection intensity and the protein-protein interactivity (data not shown).

Rigorously speaking, gene dispensability is not the biological factor to explain the degree of gene pleiotropy. Yet, gene dispensability and pleiotropy are conceptually closely related. Current understanding of genetic robustness claims that a gene is dispensable because of either genetic buffering or duplicate compensation [28]. For each mechanism, the cost for buffering or compensating a highly pleiotropic gene should also be high. Indeed, if one gene has been involved in many biological processes, genetic buffering after this gene is completely silenced may have to activate the same number of alternative pathways. Therefore, the chance for a gene to be dispensable is mainly determined by the successful probability for genetic buffering or duplicate compensation, which is inversely related to the degree of gene pleiotropy. Using over five hundred yeast single-copy genes, we have observed that the inverse relationship between *K *and the fitness after the deletion is statistically significant.

## Implications of the hypothesis

In this short article, we argue that the solution for what is the main determinant of the rate of protein evolution may depend on how to interpret the functional constraint of protein sequence. The genome factor hypothesis postulates that the functional constraint can be decomposed into a number of biological factors; but the factor-list would be endless as more functional genomics data are becoming available and more factor-factor interactions have to be considered. On the other hand, the gene pleiotropy hypothesis is to identify the degree of multi-functionality based on a statistical model of genotype-phenotype mapping, but the biological details are undetected. As they are from different routes that are not exclusive, combination of these two approaches may provide some novel insights about molecular evolution in the context of systems biology.

## Competing interests

The authors declare that they have no competing interests.

## Authors' contributions

YZ analyzed some datasets and wrote the manuscript. XG proposed the hypothesis and wrote the manuscript. All authors read and approved the final manuscript.

## Reviewers' comments

### Reviewer-1: Dr. Eugene Koonin

An interesting new look at a fundamental and certainly not completely solved problem: what determines the rate of protein evolution? Xun Gu recognizes the importance of selection for robustness to misfolding (I am not sure it is advisable to denote this effect "translational selection", a term that usually applies to selection operating at the codon choice level) but posits that functional pleiotropy is an even more critical factor. The equation (1) in this paper is helpful to lay out the dilemma explicitly: is protein evolution dominated by *B*_0 _("baseline selection intensity" which includes selection against misfolding) or by *K *(pleiotropy)? More generally, is protein evolution shaped primarily by physics of protein molecules (*B*_0_) or by function (*K*)? I certainly agree with the author of the paper that further, more careful and detailed studies of this fundamental question are required. Having said this, my current view is opposite to that expressed in Xun Gu's paper: physics is likely to be decisive. I briefly espouse two arguments.

1.	Well noticed lack of substantial correlation between "biological importance", however measured (see especially the recent thorough work of Wang and Zhang [29]). A positive correlation between pleiotropy and "importance" certainly is expected, so this does not bode particularly well for the pleiotropy hypothesis.

2.	The distribution of evolutionary rates across genome-wide sets of orthologous protein-coding genes is essentially universal among cellular life forms, from bacteria to mammals [30].

This seems to be best compatible with the hypothesis that protein evolution largely depends on the simple physics of folding (baseline selection, indeed) than on functional features of proteins like pleiotropy which hardly can be universally conserved.

*Author's response*: The central point of Dr. Koonin's criticism is that the determination of evolutionary rate could be 'physical', more precisely, the biophysical-biochemical property of proteins such as protein stability (misfoldings) or mutability. While there is no doubt that these physical properties may determine the baseline of the protein evolution, the issue is what about the variation of evolutionary rate among proteins. In the context, we have discussed substantially some issues with the first argument of Dr. Koonin. Briefly speaking, in principle we can explain why a protein is (relatively) conserved at the amino acid sequence level in some details, from physiological behavior, biological processes involved or tissue broadness. All these factors are highly heterogeneous among genes, and that is why to be a weak genomic correlation.

The second argument [also see [30]] is challenging. Indeed, the distribution of evolutionary rates across genome-wide sets of orthologous protein-coding genes is essentially universal among cellular life forms, from bacteria to mammals. This is compatible to the view that protein evolution largely depends on the simple physics of folding. However, we point out the possibility that functional features of orthologous proteins such as pleiotropy could be conserved across species, if gene co-option, an important factor in the evolution of gene regulatory network, drove the phenotypic evolution. In this case, the dimensionality of protein functionality may remain a rough constant between the orthologous genes, but the phenotypic consequences could be changed considerably. At any rate, these important speculations need to be further elaborated empirically, in spite of some preliminary analyses we already have.

### Reviewer-2: Dr. Arcady Mushegian

The paper by Gu is an attempt to juxtapose the "translational selection hypothesis", which here is stated in its extreme, and I am not sure correct, form, i.e., that the main determinant of the rate of protein evolution "is the protein stability to avoid the misfolding toxicity", and another hypothesis, namely that the main factor in protein evolution rate is its molecular pleiotropy, i.e., the ability to perform multiple functions or to contribute to multiple phenotypes.

My understanding of the translational selection hypothesis is that the major selection factor in protein sequence evolution is the minimization of misfolding burden, which is a combination of the proportion of proteins that need to be refolded or removed and of the actual toxicity to the cell of some of the misfolded variants. The first term is quite directly linked to the protein abundance in the cell, while the second may not be so.

Into this mix, the author brings in molecular pleiotropy. Unfortunately, it is not clear to me what would be the connection between the degree of pleiotropy of a protein and the protein abundance in the cell - is that a fact that actin controls more phenotypes than a repair enzyme? Furthermore, the high number of individual fitness components that may be affected by mutations in a highly pleiotropic protein does not automatically mean that the overall contribution of this protein to fitness is also higher than that of a protein with low pleiotropy.

Finally, most of the manuscript seems to restate the discussion in refs. 25 and 26 - what is the new ingredient here?

*Author's response*: We appreciate Dr. Mushegian's clarification about the translational selection hypothesis. There are two major critics. The first is about what would be the connection between the degree of pleiotropy of a protein and the protein abundance in the cell. In the revised version, we have addressed this issue, arguing that optimal functionality includes optimal protein functions and optimal expression level. Hence, a highly pleiotropic gene may require a relatively high expression level. Of course, it is only one of many mechanisms to explain the rate-expression correlation. The second critic is to question that the high number of individual fitness components affected by mutations in a highly pleiotropic protein does not automatically mean that the overall contribution of this protein to fitness is also higher than that of a protein with low pleiotropy. That is certainly true because such claim implies that the baseline selection intensity is roughly the same between the highly-pleiotropic gene and the low-pleiotropic gene. Since the baseline selection intensity can be estimated from the sequence data, the pleiotropy hypothesis can be falsified by this assumption. Therefore, we have enhanced the discussion in ref.[25,26] by explicitly formulating the hypothesis-testing framework.

### Reviewer-3: Dr. Claus Wilke

I don't understand the purpose of this paper. What exactly is the original contribution? The paper seems largely a rehashing of earlier work by the same author [25]. Maybe I'm missing something here.

Regarding the proposed hypothesis. According to the title of the paper, the pleiotropy hypothesis is presented as an alternative to the translational selection hypothesis. If this is the case, then there must be cases where the two hypotheses make different predictions, and thus experiments must (at least in principle) be able to select one hypothesis over the other. The author should list specific cases where the two hypotheses make different predictions, and they should also present at least some evidence that certain observations are better explained by the pleiotropy hypothesis than by the translational selection hypothesis. Moreover, as shown in Drummond & Wilke 2008 (ref 10), the translational selection hypothesis can explain a large set of disparate experimental observations. If the pleiotropy hypothesis is a viable alternative to the translational selection hypothesis, then it should explain the same observations at least as well, if not better. The author has to make a strong case here that their hypothesis can provide this explanatory power. Finally, the author should list a number of specific experiments or analyses that could be done in the future and that would either confirm or refute his hypothesis.

*Author's response*: We have corrected some inaccurate representations about the translational selection hypothesis in the early draft of the manuscript. We agree that gene pleiotropy hypothesis and the translational selection hypothesis are not alternative/exclusive (also see review comment-2). The real issue is their relative contributions. Though the theoretical work has been reported in our previous publications, the goal of this short article is to formulate the hypothesis that can provide the foundation for further analysis. In the revised version, we follow the suggestion to claim some potential analyses that would falsify our hypothesis.

As correctly pointed out by Dr. Koonin, the true alternative hypothesis of the gene pleiotropy hypothesis is the hypothesis of 'molecular biophysics', that is, "protein evolution largely depends on the simple physics of folding (baseline selection, indeed) than on functional features of proteins like pleiotropy which hardly can be universally conserved". The translational selection hypothesis is a special form of the later hypothesis, which may explain the rate-expression correlation. On the other hand, the gene pleiotropy hypothesis can also predict the rate-expression correlation as a highly pleiotropic gene requires, on average, high abundance of protein.
